# Serially Connected Cantilever Beam-Based FBG Accelerometers: Design, Optimization and Testing

**DOI:** 10.3390/s23063188

**Published:** 2023-03-16

**Authors:** Aarathy Ezhuthupally Reghuprasad, Chiara Colombero, Alberto Godio

**Affiliations:** Department of Environment, Land, and Infrastructure Engineering (DIATI), Politecnico di Torino, 10129 Turin, Italy

**Keywords:** fiber Bragg gratings, seismograms, cantilever-based accelerometer, geophones

## Abstract

We focus on the design, optimization, fabrication, and testing of fiber Bragg grating (FBG) cantilever beam-based accelerometers to measure vibrations from active seismic sources in the external environment. These FBG accelerometers possess several advantages, such as multiplexing, immunity to electromagnetic interference, and high sensitivity. Finite Element Method (FEM) simulations, calibration, fabrication, and packaging of the simple cantilever beam-based accelerometer based on polylactic acid (PLA) are presented. The influence of the cantilever beam parameters on the natural frequency and sensitivity are discussed through FEM simulation and laboratory calibration with vibration exciter. The test results show that the optimized system has a resonance frequency of 75 Hz within a measuring range of 5–55 Hz and high sensitivity of ±433.7 pm/g. Finally, a preliminary field test is conducted to compare the packaged FBG accelerometer and standard electro-mechanical 4.5-Hz vertical geophones. Active-source (seismic sledgehammer) shots are acquired along the tested line, and both systems’ experimental results are analyzed and compared. The designed FBG accelerometers demonstrate suitability to record the seismic traces and to pick up the first arrival times. The system optimization and further implementation offer promising potential for seismic acquisitions.

## 1. Introduction

Recently, there has been a tremendous improvement in seismic networks in terms of instrumentation and technologies providing high-resolution data [1]. Generally, based on the field of application, seismic acquisitions require instrumentation with different sensitivity and frequency ranges. Earthquake signals oscillate in a frequency range of 0.1–1 Hz; recording these signals requires high-sensitivity and low-frequency range sensors. In civil engineering, higher frequency is usually required for structural health monitoring of bridges and buildings. This instrumentation is characterized by low resonant frequency to measure the low-frequency signals generated by mechanical, active seismic sources, and the sensor should record a large bandwidth to avoid the loss of frequency components in the recorded signal and simultaneously record different seismic sources.

Existing accelerometers can be categorized into electronic and optical accelerometers. Electronic accelerometers such as micro-electromechanical (MEMS) [2], piezoresistive, capacitive, piezoelectric, and resistive sensors are commonly used [3]. The electro-mechanical accelerometers mainly consist of a mass attached to the reference frame. The working principle is based on the deflection of the proof mass due to the acceleration applied to the reference frame. Piezoelectric accelerometers are instead based on the piezoelectric response of some specific material, and they have advantages such as low cost, ease to handle and implementation due to their solid construction, wide frequency range, and temperature stability. On the other hand, they exhibit criticality because of low linearity, and under constant force, there is leakage in the charge generated on the piezoelectric element hence do not have the DC response. Piezoresistive accelerometers are suitable for low frequency, small volume, low linearity, and measuring static as well as dynamic acceleration (DC response) but could be sensitive to temperature. Capacitive accelerometers are low cost, have low power consumption, high sensitivity, and DC response but interfere with the external environment and suitable for low-frequency fields. All these systems are vulnerable to electromagnetic interference because of their sensing principle [4].

The optical accelerometers are mainly based on fiber optic sensing technology. Fiber optic sensing techniques such as distributed acoustic sensing (DAS) [1,5,6,7], fiber Bragg grating (FBG) [8,9,10], and fiber Fabry-Perot interferometer (FFPI) [11,12,13,14,15] have been extensively studied for vibration measurements in recent decades. Compared to the other accelerometers, they are immune to electromagnetic interference. They also show high sensitivity, compactness, capability to transmit signals at long distance, DC response, wide bandwidth, corrosion resistance, high temperature, and pressure resistance. Competing with other optical accelerometers, fiber Bragg grating-based sensors have undergone rapid development and established themselves as the leading technology for onsite measurements in structural monitoring, for vibrational acceleration measurement of low-medium frequency ranges with high sensitivity.

Various cantilever beam-type accelerometers of fiber Bragg grating have been reported in the past decades. In 2009, Wu et al. analyzed the potentialities of three different FBG-based accelerometers for the study of different seismic measurements [16]. Later, Costa Antunes et al. proposed an L-shaped cantilever beam accelerometer suitable to monitor frequencies up to 45 Hz [17]. A compact FBG diaphragm accelerometer with L-shaped and U-shaped rigid cantilever beam, with a wide frequency range and high sensitivity, was reported by Weng et al. [18,19]. An alternative approach was developed by Basumallick et al. to enhance the sensitivity (450 pm/g) by increasing the distance between the neutral axis of the fiber and the axis of the fiber using a backing patch [20,21]. Feng et al. demonstrated a hybridized two-cantilever FBG accelerometer with temperature compensation and minimum cross-axis sensitivity [22]. Casas Ramos et al. put forward a sensor design by improving the resonant frequency without affecting the sensitivity [23,24,25]. Guo et al. suggested the fabrication of a vibrating element in the FBG accelerometer with 3D printing technology [26]. Zhang et al. proposed a double semi-circle cantilever beam with high sensitivity and a minimum detectable frequency of 5 Hz [27]. Hong et al. designed a multi-cantilever beam mainly composed of three rectangular cantilever beams with low frequency, within the range of 16–54 Hz and a sensitivity of 87.955 pm/g [28]. A low-frequency FBG accelerometer based on a polyphenylene ether (PPE) thermoplastic cantilever beam with a sensitivity of 110 pm/g and a natural frequency of 9 Hz was proposed by Hafizi et al. [29].

However, the reported literature studies on FBG accelerometers are mainly concentrated on the design, fabrication, and experiments in the lab. There remains a need for the efficient study of the reliability of these systems in terms of packaging and installation for practical applications, especially in real field cases.

Aiming at the problem of reliability of these systems in the practical applications, we design, optimize, and test this cantilever beam-based accelerometer in the field in comparison with the standard geophones; the main goal was to check the suitability of the proposed sensors for picking up the first arrival time (travel times), which is a common value estimated in seismic exploration. This accelerometer was theoretically and experimentally analyzed in terms of sensitivity and natural frequency; moreover, the theoretical simulations and design optimizations were undertaken with the ANSYS software. The proposed design is fabricated with the help of 3D printing technology and packaged in a cost-effective way. Experimental results from the laboratory show that the sensor has a flat frequency response from 5 to 55 Hz. The sensitivity of the accelerometer is ±433.7 pm/g with a natural frequency of 75 Hz. The preliminary test performed in the field in comparison with common electro-mechanical geophones for picking up the first arrival time was successful in verifying its effectiveness in seismic vibration measurements. 

## 2. Materials and Methods

### 2.1. System Description

Fiber Bragg gratings (FBGs) are formed by the periodic modulation of the refractive index along the longitudinal direction of the photosensitive single-mode fiber core [30]. The peak wavelength reflected by the FBG is the Bragg reflection wavelength and is mainly dependent on the effective refractive index of the fiber core and the period of the grating. Any change in the effective refractive index or the grating period will cause a shift in the Bragg wavelength. This shift can be used to measure various physical parameters, such as temperature, strain, etc. Typical value of strain and temperature sensitivity of standard FBG sensors are 1.2 pm/με (με = micro strain) and 13 pm/℃ respectively [31]. These FBG sensors are sensitive to both strain and temperature variations directly, but can indirectly be exploited to quantify other physical parameters, such as pressure, displacement, and vibration by using transducers, e.g., specifically designed elastic structures, to relate them to axial strain [9].

**Figure 1:**
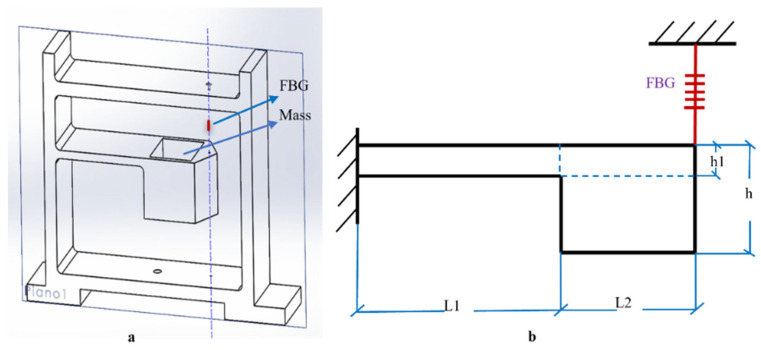
Structure of the cantilever-based FBG sensor: (**a**) system 3D rendering generated by Solid Works (2020); (**b**) schematic section of the cantilever.

We propose an FBG cantilever beam-based accelerometer that could be customized to adjust sensitivity and resonant frequency depending on the scope of the measurements. The mechanical structure of the accelerometer is a single cantilever beam with mass block at its tip suspended at a certain height within an encasing structure. FBG is positioned and fixed perpendicular to the surface of the vibrating cantilever beam through a hole made in the encasing structure. The schematic diagram of the proposed cantilever mass-based accelerometer is presented in Figure 1. Here FBG is used as a spring that enhances the natural frequency of the cantilever and uniformly strains the grating to increase the sensitivity of the system to vibrations. Pretension is applied for the FBG by gluing two points at the end of the cantilever beam and the encasing structure with a white epoxy adhesive. This epoxy is mainly used in structural bonding applications for high chemical and temperature resistance.

When a vibration is applied at the base of the structure, the acceleration induces a force on the mass block resulting in the deflection of the cantilever beam. The vertical deflection of the cantilever beam elongates the gratings of FBG resulting in the Bragg wavelength shifts due to strain. This corresponding bending strain is proportional to the vertical acceleration. Hence this system is more sensitive to recording vibrations along the vertical axis. The response and resonance along the other two components are consequently not taken into consideration in this study.

The natural frequency and the sensitivity of the proposed design are increased by placing the FBG sensor at the maximum bending point, which uniformly strains the FBG axially with the vertical axis. The sensitivity, operational bandwidth, and natural frequency of the proposed design can be tailored by changing the cantilever beam parameters (length, breadth, and thickness), weight of mass block, material of the cantilever beam and mass block, which depends on the young’s modulus and density of the material.

### 2.2. Preliminary Test

A preliminary lab test was carried out on an FBG sensor having the same design described in Cavalli [32], having a (theoretical) resonance frequency of 128 Hz and sensitivity of 956 pm/g. The sensor structure consists of a cantilever beam with a Cantilever length (L1) of 30 mm, a mass block length (L2) of 20 mm, total length of cantilever beam (L = L1 + L2) of 50 mm, breadth of 20 mm, thickness (h1) of 5 mm (Figure 1b); a block mass of lead (88 g) is inserted at the tip of the cantilever beam. The whole system is fabricated with polylactic acid (PLA) by 3D printing. The elastic modulus and the cross-section of the fiber optic SMF-28 with protective coating are considered from Antunes [33,34]. The FBG used in this system has a central wavelength of 1538.12 nm, reflectivity ≥ 90%, and length of 10 mm. This sensor was spliced to the fiber patch cord and connected to the SMART SCAN Interrogator for data acquisition with a sampling frequency of 500 Hz. This is a wavelength division multiplexing instrument with a tunable laser source that enables high resolution at multi-kHz frequencies and consists of four optical channels; hence, multiple sensors can be multiplexed without reducing the speed and performance.

**Figure 2:**
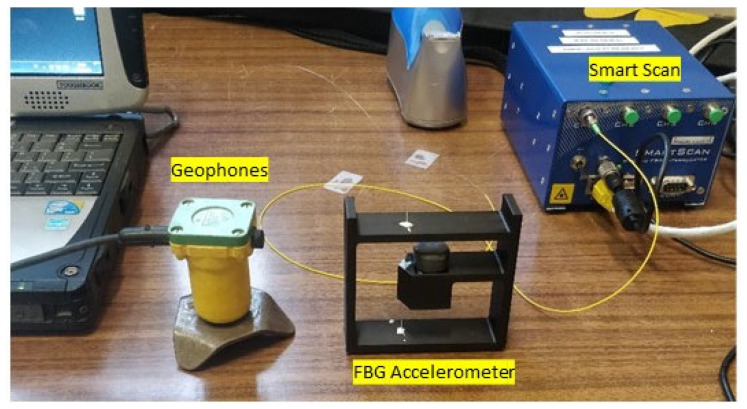
Comparison test between the FBG accelerometer and a standard vertical 4.5 Hz geophone in the laboratory with external hammer shots.

We compared this FBG cantilever system with a common 4.5-Hz vertical geophone used for active seismic recording (Figure 2), connected to a Geode Exploration Seismograph (Geometrics). A light hammer was used to generate vibrations to be recorded by both instruments. The geophone system was automatically triggered (time = 0 s corresponds to the impact of the hammer), while the recording of the FBG system was manually triggered by the operator during the test.

**Figure 3:**
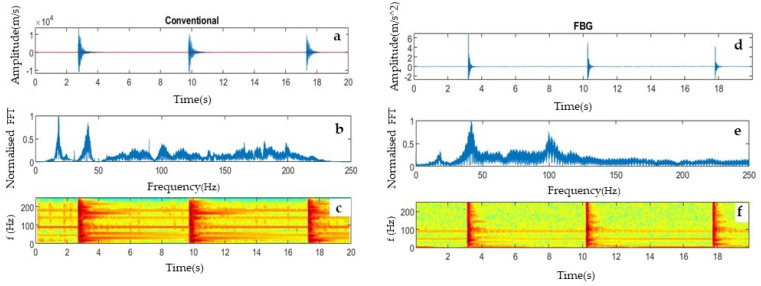
Exemplificative comparison of time- and frequency- domain signals from the conventional geophone (**left**) and the FBG system (**right**) in the preliminary laboratory test. (**a**) Amplitude of the signal of the conventional geophone in the time domain; (**b**) normalized amplitude spectrum, (**c**) spectrogram, (**d**) amplitude of the signal of FBG accelerometer in the time domain; (**e**) normalized amplitude spectrum, (**f**) spectrogram.

Data recorded from both systems were analyzed with on-purpose compiled MATLAB codes to compare their time- and frequency-domain content. In Figure 3, exemplificative signals (Figure 3a,d), related normalized amplitude spectra (Figure 3b,e), and spectrograms (Figure 3c,f) are shown for the FBG accelerometer and the standard geophone, respectively. A time shift can be noticed between the first signal recorded by the two systems (Figure 3a,d) as a consequence of the lacking triggering system for the FBG sensors. Nevertheless, the time delay of the following signals with respect to the first one is the same for both systems.

The results from the preliminary test showed promising potential for recording seismic vibrations. Nevertheless, the FBG sensor recordings showed lacking information related to the first signal onset. Using the spectral amplitude of the geophone as a reference, the FBG sensor showed lower sensitivity in the low-frequency range, below 20 Hz. Similar spectral response is detected by both instruments, but with different amplitudes. These preliminary results highlighted the need for numerical optimization of the system configuration for improving the sensitivity of the system in the low-frequency range. The accelerometer design, which was further optimized by theoretical simulations using ANSYS finite element modeling software, is reported in Section 2.3.

### 2.3. Numerical Simulations of the Effect of Structural Parameters on the Natural Frequency

The length, breadth, and height of the cantilever beam are the key parameters affecting the sensor sensitivity S and natural frequency F_0_. The natural frequency of the sensor is mainly ruled on the possible measuring range and the vibration performance of the sensor. To study the effect of the system geometry on the natural frequency, cantilever beams with various dimensions were modeled, and their response was simulated with the finite element modeling software ANSYS Student (2023).

Modal analysis is an analytical method for determining the vibration characteristics of the design. In this analysis, we obtain the natural frequencies and modes of the different sensor designs. Here, the modal analysis of the design was conducted to calculate, solve, and extract the natural frequency of the first six vibration modes. The system structure was printed with PLA, so the material properties adopted in the simulation were easily user-defined with the parameters summarized in Table 1. The block mass (20 g) inserted at the tip of the cantilever is made of lead.

During the simulations, the base of the system (Figure 1a) was set as a fixed constraint to consider the coupling with the ground surface. The remaining domains and boundaries were left free to vibrate. The displacement of the beam was set free in the vertical direction and constant in the other two directions.

**Figure 4:**
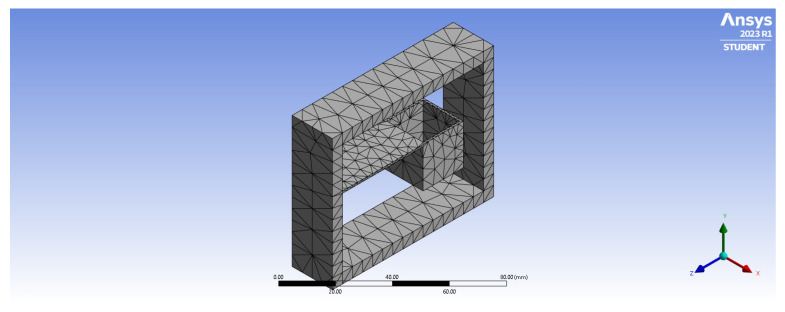
Mesh of the cantilever-based FBG accelerometer.

In the simulation (Figure 4), we have considered cantilever lengths (L1 in Figure 1b) within the range of 30–45 mm at three different heights (1 mm, 3 mm, and 5 mm, h1 in Figure 1b) by keeping the cantilever’s breadth constant (20 mm). A total of twelve configurations were consequently simulated and analyzed for the estimation of the natural frequency of the system.

### 2.4. Lab Calibration of the System

**Figure 5:**
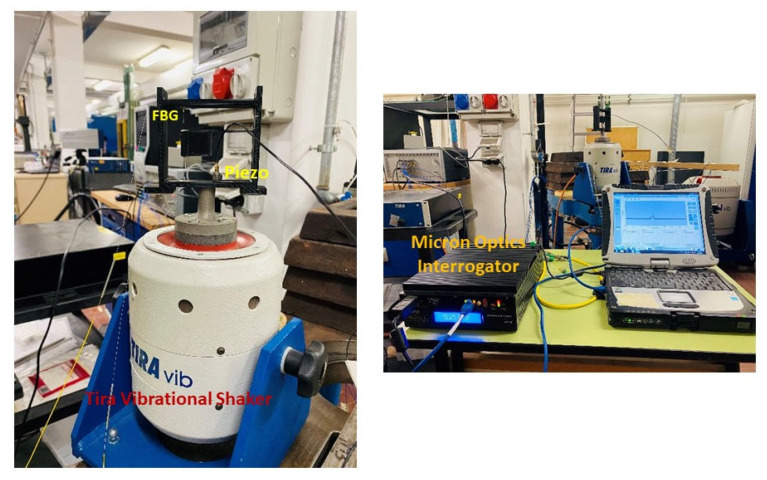
Laboratory calibration test of FBG system with a mechanical shaker.

After numerical simulations, four system configurations were selected for laboratory calibration, as summarized in Table 2. Figure 5 shows the schematic diagram of the vibrational shaker set up with the prototype FBG accelerometer and vibration exciter. The amplitude frequency response test is conducted by attaching the base of the FBG accelerometer to the Tira vibrational shaker using glue. The vibrational shaker provides a linear oscillatory motion in the vertical direction. This device is paired with a function generator and an amplifier. The input source of the vibrational shaker is a sweep signal (harmonic sinusoidal) with a sweep frequency ranging from 5 Hz to 110 Hz at a peak acceleration of 0.1 g at intervals of 5 Hz with a time lag of 10 s at each frequency. Near the simulated resonance frequency, the outputs are recorded at a smaller interval of 1 Hz for tracking the exact natural frequency. A Micron Optics Si155 interrogator system was used to acquire the data of FBG wavelength shift with a sampling frequency of 1 kHz. This system can potentially acquire the measurements of hundreds of sensors on four parallel and 160 nm wide optical channels.

The dynamic sensitivity at different frequencies was tested by varying the Vpp (peak-to-peak voltage) from 0.2 to 1 with the step size of 0.2, with the frequency of the vibration of the shaker set to 20 Hz. We repeated the same experiment at frequencies of 10, 30, and 40 Hz. A piezo-electric accelerometer with a frequency response from 0 to 20 kHz was additionally placed on the base of the system as a reference measure without altering the cantilever response. The sensitivity was calculated by dividing the wavelength shift from the data measured by Micron Optics Interrogator by the corresponding acceleration observed by the piezo accelerometer. 

## 3. Results

**Figure 6:**
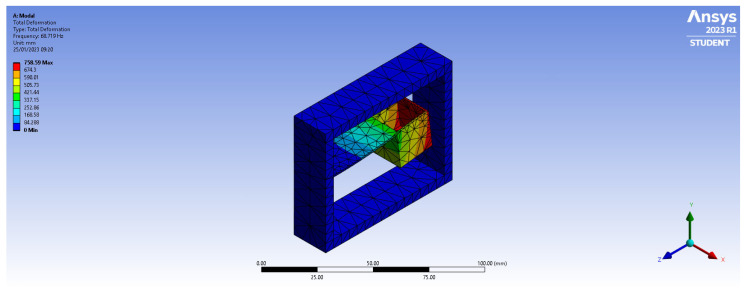
First vibration mode in modal analysis for system configuration having cantilever length of 40 mm, thickness of 1 mm, and breadth of 20 mm.

The primary mode of vibration from the ANSYS Modal analysis is shown in Figure 6 for one of the simulated configurations (L1 = 40 mm, thickness 1 mm, and width 20 mm). The resonance frequencies of the first six orders are reported in Table 3 for the same system configuration. 

**Figure 7:**
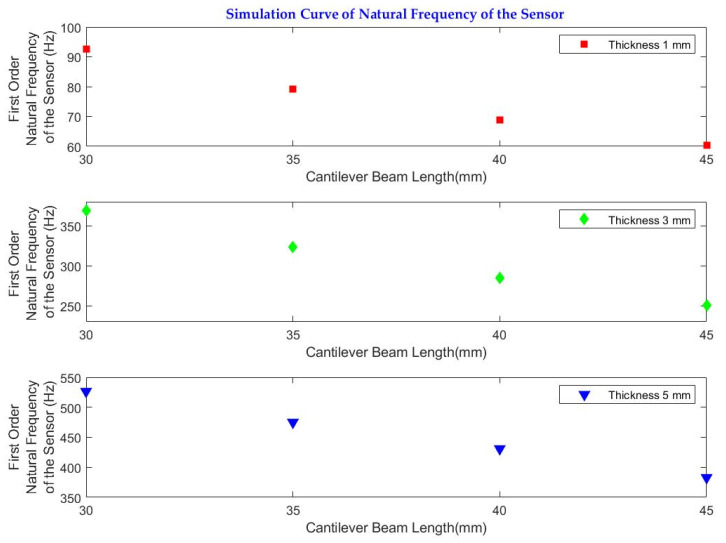
Sensitivity analysis of natural frequency values for different cantilever beam lengths and thicknesses.

As shown in Figure 7, the simulated natural frequency of the system decreases with the increase in the cantilever beam length from 30 mm to 45 mm. In systems having the same cantilever beam length, the natural frequency increases and with the increase in the cantilever beam thickness.

Based on the results of the sensitivity analysis, the cantilever configuration having the lower natural frequency (68.7 Hz) was selected to be printed and further tested in the lab and field experiments. The geometry and structure of the selected configuration are summarized in Table 4.

**Figure 8:**
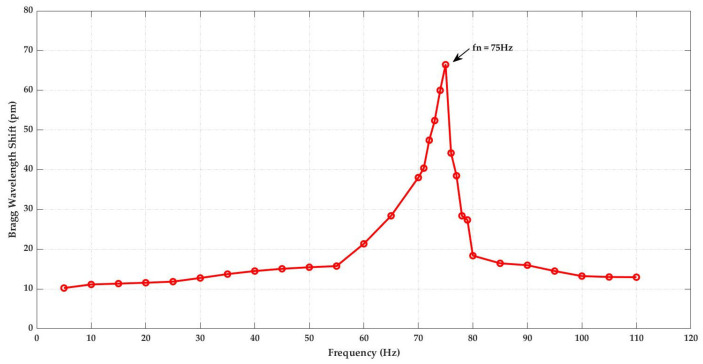
Amplitude-frequency response of the FBG accelerometer.

For this system configuration, the amplitude-frequency response of the accelerometer retrieved from the laboratory calibration on the vibrational exciter(shaker) is 75 Hz (Figure 8). The accelerometer shows an almost flat response within the range of 5–55 Hz, so being applicable for the measurement of low-frequency signals. The theoretically calculated natural frequency (68.7 Hz) for this system configuration is close to the measured one (75 Hz). Minor discrepancies can be due to the mechanical losses during the gluing of the sensor to the shaker.

**Figure 9:**
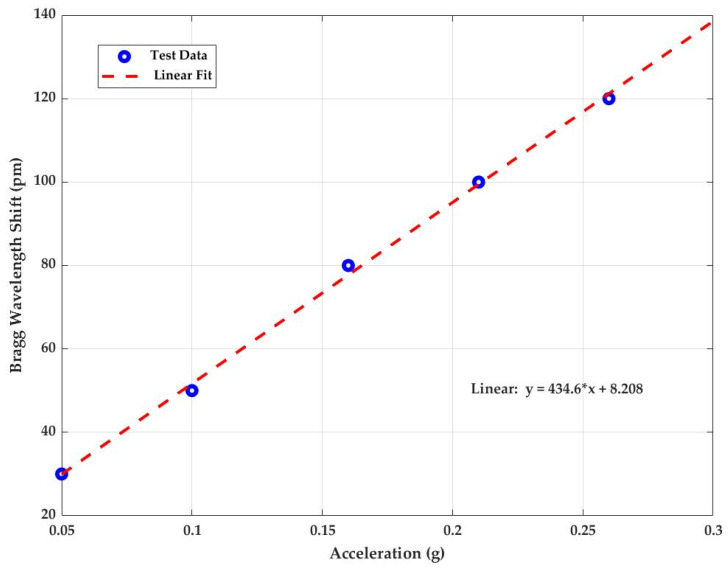
Linear response of Bragg wavelength shift versus applied acceleration at frequency 20 Hz.

The dynamic sensitivity of the accelerometer was tested at room temperature. The wavelength shift corresponding to different g values at 20 Hz is shown in Figure 9 as dots, and the dashed line represents the linear fit. The slope of the linear fit gives the sensitivity of the sensor. The sensitivity of the sensor calculated from this experiment with fit coefficient R^2^ = 0.998 at 10 Hz, 20 Hz, 30 Hz, and 40 Hz are ±435.1 pm/g, ±434.6 pm/g, ±409.3 pm/g and ±455.8 pm/g. The average sensitivity of this FBG accelerometer calculated from this experiment is ±433.7 pm/g with a fit coefficient R^2^ = 0.998.

## 4. Field Test of the Calibrated System

A preliminary field test was carried out with the calibrated FBG accelerometer from the FEM simulation and vibration exciter experiment. The final geometry has a length (L1 + L2, in Figure 1a) of 60 mm, a breadth of 20 mm, and a thickness (h1) of 1 mm. The cantilever is inserted in a structure with a size of 80 mm × 65 mm and suspended at a high of 20 mm (Figure 1a). The packaging of the system is performed by encasing the system inside a PVC box with two spikes attached at the bottom of the box to improve the coupling with the ground. Three FBG sensors with this configuration were used for the field test, with FBG length of 10 mm, central wavelengths of 1532.45 nm, 1538.65 nm, and 1546.12 nm, and reflectivity ≥ 90%.

**Figure 10:**
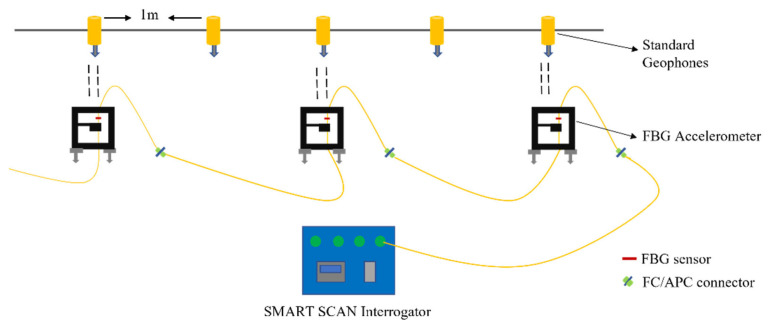
Schematic diagram of the field test.

**Figure 11:**
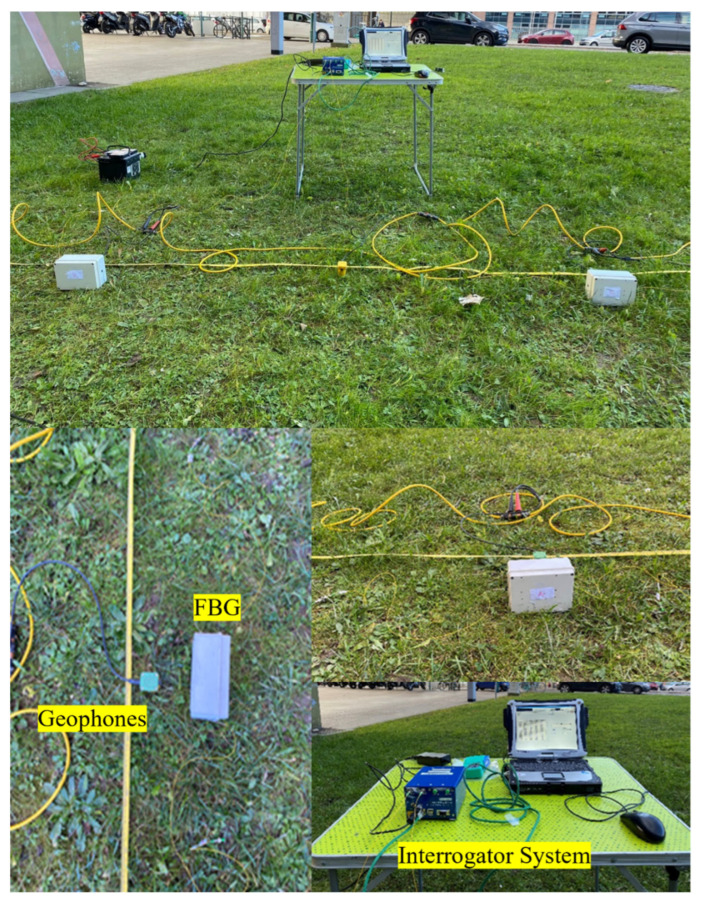
Field test of the calibrated system with the parallel standard geophones.

The field test was conducted to compare the three serially connected packaged FBG accelerometers with five parallelly connected 4.5-Hz vertical geophones, as shown in Figure 10 and Figure 11. The 4.5-Hz vertical geophones were used as a benchmark to evaluate the performance of the FBG accelerometer. A sledgehammer impinging on a metallic plate was used to generate shots at the two edges of the line, at 1 m from the first and last sensors. A trigger delay of 0.1 s and a 10-shot stacking was set for the standard geophone acquisition, while FBG traces were acquired continuously due to the lack of synchronization between the two systems. Similar to the previously described lab test, the recordings were analyzed using on-purpose written MATLAB codes to visualize time and frequency signal features.

**Figure 12:**
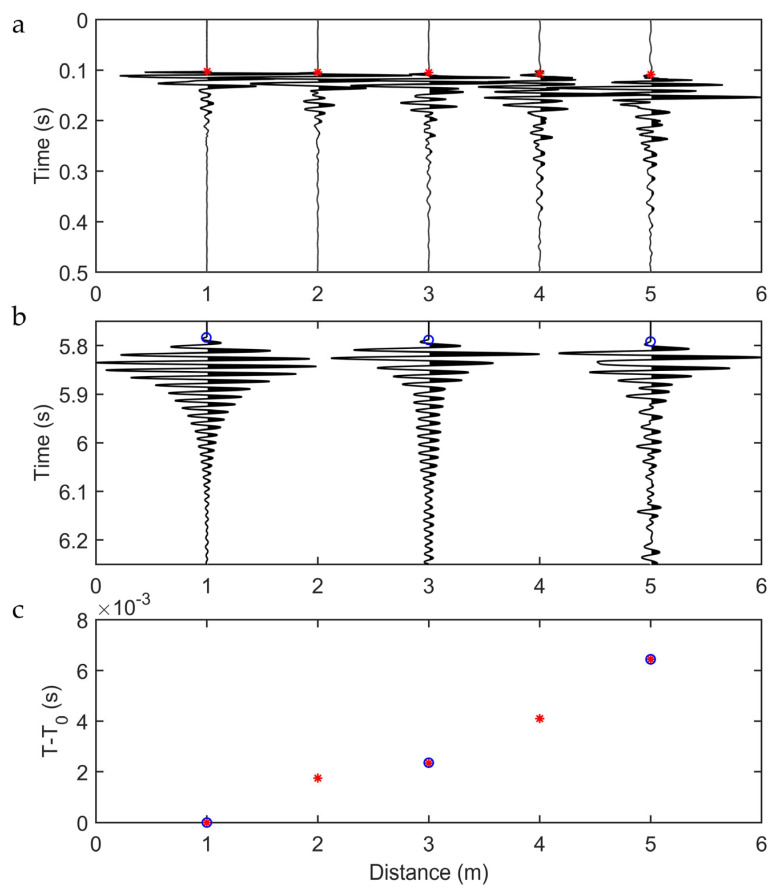
Seismograms of (**a**) standard geophones and (**b**) FBG accelerometer for the external hammer shots, (**c**) first arrival times in terms of delta T in the *y*-axis with respect to the sensor position of both systems.

The recorded seismic traces from both instruments are shown in Figure 12a,b. Since the FBG system lacks an automatic triggering and stacking system, trace stacking to improve the signal-to-noise ratio was implemented by means of cross-correlation. In particular, the continuous FBG recordings were split into shorter segments, roughly containing the traces related to each hammer shot. Cross-correlation between the trace closest to the source of the first shot segment and each following time segment was then undertaken. The obtained lag (delay) computed between the different time windows was then used to align the time segments and stack the traces.

In Figure 12a,b, each geophone and FBG trace is normalized to its maximum amplitude, so no information on the attenuation of the signal amplitude can be inferred from these plots. However, the FBG sensors carry a signal with different amplitude distributions in time after the first arrival time. This is mostly due to the different amplitude in frequency bands recorded by the two sensors. However, these factors did not affect the first arrival time reading.

First arrival time picking was then carried out on the five stacked geophone traces and the three stacked FBG traces. Due to the missing triggering system, the time picked at the first sensor (i.e., closest to the source) was removed from both datasets. The related delay times at the different sensors are shown in Figure 12c. The plot shows almost perfect matching between the picked times, demonstrating the system improvement with respect to the preliminary lab experiments.

## 5. Discussion and Conclusions

We designed and tested a simple cantilever beam-based FBG accelerometer suitable for picking up the first arrival time of the active seismic source in the field. Using the already established fiber optic sensing technique, we designed, developed, fabricated, and packaged an FBG-based accelerometer. For field purposes, these accelerometers are serially multiplexed and directly coupled to the ground, just like standard vertical geophones. Using a single interrogator, some decades of sensors could be serially multiplexed and connected to the same fiber.

Initially, we carried out an FEM simulation using ANSYS to decide the measuring range (bandwidth) and performance, which is associated with the natural frequency of the sensor. The key parameters affecting the natural frequency of the designed sensor are the length, thickness, and breadth of the cantilever beam along with the mass block. In the simulations, the breadth of the cantilever beam (20 mm) is kept fixed, and the cantilever beam length (from 30 mm to 45 mm) and thickness (from 1 mm to 5 mm) are varied to study the effects of parameters on the natural frequency. This analysis was useful to check the properties of the sensor with respect to the expected sensitivity and frequency response.

Further, we calibrated the simulated design configurations in a laboratory using a vibrational exciter by performing the amplitude-frequency response and dynamic sensitivity test. With the help of Solid Works, the 3D model of the design was generated and fabricated using the 3D printing technology with PLA for the laboratory calibration test. The 3D printing technology was a time saver compared to other production facilities; it helped to do more for the customization of the system due to the fast production and the cost. The test results are found to closely match with ANSYS modal analysis and lab calibration. The optimized FBG accelerometer (with the following cantilever beam parameters: total length = 60 mm, breadth = 20 mm and thickness = 1 mm, mass block of lead = 20 g) has a natural frequency of approximately 75 Hz, with an operating frequency range of 5–55 Hz, where the response remains almost flat and has a high sensitivity of ±433.7 pm/g. Overall the performance achieved for the simple and compact 3D-printed proposed sensor is best for low-frequency measurements with high sensitivity.

To test these 3D-printed devices in the field, we packaged the sensor inside a PVC box with two spikes attached at the bottom of the box to improve the mechanical coupling with the ground. This packaging approach makes it relatively compact, lightweight, and robust for field applications. From the field test in comparison with the 4.5 Hz vertical geophones, we observe that the first arrival time of both systems is perfectly matching. Hence, this sensor system has the potential to monitor active signals.

One of the limitations is the fragility of the FBG sensors, so the installation of these sensors should be taken care well than the conventional ones, which are more robust. Despite this limitation, the multiplexing capability of the FBG sensors, immunity to electromagnetic interference, and the capability of the interrogation system to accommodate more sensors in one go add up to the advantage of using such systems instead of the conventional ones. In the lab and field tests, we used two different interrogators maintaining the same acquisition parameters. Both demonstrated suitable recording of the input vibrations of the designed system.

The prime objective of this work was to introduce a simple and relatively compact FBG cantilever beam-based accelerometer design with low-cost fabrication and packaging methods for monitoring active signals in the field; still, there is enough scope for further improvement. The advantage of this work with respect to existing designs is flexibility in reproducing and optimizing the design due to the ease of fabrication and low-cost packaging. In this way, the FBG system can be easily customized depending on the desired application. Since we have not performed the temperature compensation with a reference fiber in the system, it may fail in an operating environment with significant temperature variation. This aspect will be further addressed in future studies. We still have room for improvement in improving the robustness of these systems and developing the triggering system for data acquisition.

## Figures and Tables

**Table 1:** Material properties adopted in the simulations.

Physical and Mechanical Parameters	PLA	Lead
Young’s Modulus (Pa)	4.1 × 10^7^	5.5 × 10^6^
Density (kg/m^3^)	1290	9670
Poisson’s Ratio (-)	0.3	0.4

**Table 2:** Configuration of the systems used for lab calibration.

	Cantilever Length (mm)	Cantilever Thickness (mm)
1	30	1
2	30	3
3	40	1
4	40	3

**Table 3:** First six resonance frequencies (Hz) obtained for the system configuration of Figure 6.

Order	First	Second	Third	Fourth	Fifth	Sixth
Resonance Frequency (Hz)	68.7	212.8	415.8	684.2	941.9	1271.0

**Table 4:** Cantilever beam configuration for laboratory and field tests.

Parameter	Value
Length of cantilever (L1)	40 mm
Mass Block length (L2)	20 mm
Total Length of Cantilever (L = L1 + L2)	60 mm
Width of the cantilever beam	20 mm
Thickness of the cantilever beam	1 mm
Fiber length from enclosure to the cantilever tip	20 mm
Lead mass	20 g

## Data Availability

Data of the laboratory and field tests carried out in this study are available upon request to the corresponding author.
